# Knowledge, attitudes and practices among healthcare professionals regarding complementary alternative medicine use by patients with hypertension and type 2 diabetes mellitus in Western Jamaica

**DOI:** 10.1016/j.ctim.2021.102666

**Published:** 2021-01-15

**Authors:** Grace Kwak, Kimberly Gardner, Bolanle Bolaji, Sarah Franklin, Maung Aung, Pauline E. Jolly

**Affiliations:** a Department of Epidemiology, School of Public Health, University of Alabama at Birmingham, Birmingham, AL, USA; b Epidemiology and Research Unit, Western Regional Health Authority, Ministry of Health and Wellness, Montego Bay, St James, Jamaica

**Keywords:** Complementary and alternative medicine use, Healthcare professionals, Hypertension, Type 2 diabetes mellitus, Chronic diseases, Jamaica

## Abstract

**Objectives:**

This study investigated the knowledge, attitudes, and practices (KAP) of healthcare professionals (HCPs) regarding CAM use for Hypertension (HTN) and Type 2 Diabetes Mellitus (T2DM) among patients in western Jamaica, and to determine HCPs’ perceptions of the need for training on CAM.

**Design:**

A cross-sectional study was conducted from May to August 2019.

**Setting:**

HCPs serving patients with HTN and T2DM in chronic disease clinics in western Jamaica completed a self-administered questionnaire that provided data on their sociodemographic characteristics, training, and KAP of CAM.

**Main outcome measure:**

The data identified factors associated with discussion and recommendation of CAM to patients and personal use of CAM by HCPs.

**Results:**

Type of profession (physicians vs nurses OR = 2.17; 95 % CI = 1.07–4.42 and pharmacists vs nurses OR = 8.67; 95 % CI = 2.83–26.57) was significantly associated with discussion of CAM. Training on CAM was significantly associated with discussion (OR = 2.36; 95 % CI = 1.26–4.42), recommendation (OR = 2.72; 95 % CI = 1.36–5.42), and personal use of CAM (OR = 2.90; 95 % CI = 1.69–4.97). Dieticians and nutritionists had 4.56 higher odds of personal use of CAM (95 % CI = 1.16–17.86), and personal use of CAM was significantly associated with discussion (OR = 8.94; 95 % CI = 4.76–16.80) and recommendation (OR = 7.17; 95 % CI = 3.54–14.51) of CAM. The majority of HCPs (70–89 %) agreed that there is a need to include CAM in professional training programs.

**Conclusion:**

The results of this study can be used to guide development of programs for training HCPs on knowledge and safe use of CAM so that they can better serve their patients.

## 1. Introduction

In 2017, stroke and diabetes mellitus (DM) were the two leading causes of mortality in Jamaica, and DM was the leading cause of disability.^1^ Approximately 30.8% of the population of Jamaica is hypertensive and 11.4% is diabetic.^2,3^ Thus, roughly 1.2 million of the total population of 2.91 million Jamaicans have either hypertension (HTN) or DM.^4^ Prior evidence of diabetes was found to be associated with a twofold to threefold increased risk of clinical atherosclerotic disease. Therefore, treating these diseases effectively is key to controlling the development of further disease.^5^ A study on the quality of control and adherence to HTN treatment prescribed for 756 Jamaicans showed very low adherence to the treatment.^6^

Globally, healthcare professionals (HCPs) use conventional medicine for management of HTN or DM; however, there is high prevalence of use of complementary and alternative medicine (CAM) for managing these diseases.^7^ CAM has been defined as a group of diverse medical and healthcare interventions, practices, products, or disciplines that are not generally considered part of conventional medicine.^8^ A study conducted in 15 countries found that the prevalence of CAM use ranged from 9.8%–76%.^7^ The type of CAM used varies across regions. A study conducted in the United States found prevalent therapies included herbal medicine, massage, megavitamins, self-help groups, folk remedies, energy healing, and homeopathy, while studies in Jamaica and Trinidad and Tobago found that herbal medicine was the predominant CAM intervention.^9–15^ Energy Healing is a form of healing that manipulates, restores or balances the flow of energy in the body. The energy is channeled through the practitioner to the client, helping to remove energy deficiencies and blockages, which then activates the body’s own natural ability to heal itself.^16^

There is a high prevalence of concomitant herb-prescription drug use among Jamaicans, especially among those of low socioeconomic status and those without health insurance.^17^ In a study on CAM use among patients with HTN and/or Type 2 DM (T2DM) in western Jamaica, we found that 79% of those with HTN and 65% of those with T2DM reported current use of CAM, and 60–70 % used CAM simultaneously with prescription medicine.^18^ In the same study, only about 20 % of participants reported discussing CAM use with their HCPs. Other research shows that low percentages of HCPs are aware of herbal use by their patients.^19^ This could be attributed to the fact that many patients expect negative reactions from HCPs about their CAM use and prefer not to disclose such information.^20^

A study conducted on KAP of CAM among physicians in Trinidad and Tobago found that 60% of the physicians believed that herbal remedies were beneficial to health.^14^ These physicians had relatively high acceptance, but poor knowledge, of these herbal remedies; only 15% were able to identify at least one known herb-drug interaction. Another study from Trinidad and Tobago found high prevalence of CAM use among HCPs.^21^ The majority (50–75%) of these HCPs reported that they had fair knowledge of CAM and while about half said they were likely to ask their patients about CAM, less than 15% were likely to refer patients to a CAM practitioner.^21^ However, 50–60 % of these HCPs felt that combination therapy is superior to conventional medicine alone.

Many patients do not feel comfortable discussing their CAM use with their HCPs despite concerns about drug interactions between CAM and prescription medicines.^17,22–25^ A study conducted in Jamaica examining herbal medicine use in conjunction with conventional medicine found that only 18% of participants reported that their HCPs knew of their herbal medicine use and most said that they voluntarily told their HCPs; only 11.3% reported that their physicians asked about their herbal medicine use.^17^ This shows a significant lack of communication about CAM between patients and physicians in Jamaica. Some patients reported that when they asked their pharmacist about the possible adverse effects of CAM use with prescription medication, the pharmacist often did not give a sufficient answer or was unable to answer the question.^17,22–25^

This study is the first that has been conducted to determine KAP among HCPs (doctors, nurses, nutritionists, dieticians and pharmacists) in western Jamaica regarding CAM use for HTN and T2DM among patients and to determine HCPs’ perceptions of the need for inclusion of CAM in curricula during professional training.

## 2. Methods

### 2.1. Study design, site, and study population

We conducted a cross-sectional study from May to August 2019 among HCPs working in any of the 4 parishes under the Western Region Health Authority (WRHA); namely, Hanover, St James, Trelawny, and Westmoreland western Jamaica. The WRHA covers 3,939km^2^ area with an approximate population of 600,581. Eligible participants were HCPs (clinicians, nurses, pharmacists, dieticians and nutritionists) ≥18 years of age who worked in Curative Clinics caring for patients with HTN and/or T2DM. HCPs who did not satisfy the eligibility criteria were excluded from the study.

### 2.2. Sample size and participant recruitment

Based on a previous estimate of the proportion of HCPs (50%) who self-reported fair knowledge of CAM in Trinidad and Tobago, the sample size for the current study was determined to be a minimum of 235 HCPs with a 95% confidence limit and 5% margin of error.^20^ The sample was calculated using the online calculator EpiTools by Ausvet© (http://epitools.ausvet.com.au). HCPs were informed of the study by the regional Coordinator of Chronic Non-Communicable Diseases Programme for the WRHA and were introduced to the research staff by their department heads at staff meetings. The research staff provided information on the study and answered questions from the HCPs. Signed informed consent was obtained from each HCP who volunteered to participate.

### 2.3. Questionnaire development and data collection

A 49-item self-administered questionnaire was used to gather information on sociodemographic factors (age, sex, parish, marital status, religion), training of HCP, years of experience, training in CAM, KAP of CAM, and personal use of CAM. Questions on knowledge and perception of CAM and concomitant use of CAM with prescription medicines were adapted from questions used in published studies on CAM that were conducted in Jamaica and Trinidad and Tobago.^14,17,21,26^ The questions used on the CAM knowledge test were developed from systematic reviews of published papers on the effects of herbal treatments especially garlic on lipid profile and glucose parameters in diabetic and cardiovascular disease patients and on the anti-inflammatory and blood-thinning effects of ginger.^27–30^ This test had 9-items requiring True or False answers as follows: 1) There are several completed and ongoing international clinical trials on the efficacy and safety of herbal medicines; 2) Each herb usually consists of a single potentially active pharmaceutical constituent; 3) Herbs do not work synergistically with prescribed medicines; 4) Some herbs inhibit the effect of prescribed medicines; 5) Interaction between some prescribed drugs and herbal medicines can be harmful to patients; 6) Garlic preparations have not been found to be effective in reducing blood pressure in patients with HTN; 7) Ginger has been shown to improve blood circulation and relax the muscles surrounding blood vessel; 8) Garlic interacts with some diabetic drugs to result in hypoglycemia; 9) Garlic has not been shown to have anti-oxidative properties. The questionnaire was reviewed by Jamaican health officials and revised. It was then pilot tested among 8 HCPs similar to those recruited for the study and revised before use.

### 2.4. Data analysis

Data analysis was conducted for 288 HCPs (144 doctors, 108 nurses, 12 dieticians/nutritionists, 27 pharmacists) who met the inclusion criteria and completed the questionnaire. Family nurse practitioners were combined with nurses because of the small number in the former group. Dieticians and nutritionists were also combined into one group because of small numbers in each group. Descriptive analyses comprising frequencies and percentages for categorical variables and means for continuous variables were computed. Knowledge scores were computed for the HCPs based on the sum of correct responses to the nine True/False questions on CAM. Knowledge scores were modelled as a categorical variable, low (0–3), average (4–6) and good (7–9).

Characteristics across HCPs categories were compared using chi square for categorical variables and *t*-test or ANOVA for continuous variables to test for significant differences. We examined the association between demographic and other factors, and outcomes, which included discussing CAM use with patients, recommending CAM use, and personal use of CAM. Covariates that were significantly associated with HCPs and outcomes for a p-value <0.05 were included in multivariable logistic regression analyses to compute estimated odds ratio (OR) and 95% confidence intervals (CI) to identify factors significantly associated with discussion of CAM use, recommendation of CAM and personal use of CAM. Data analyses were conducted using Statistical Analysis Software (SAS) version 9.4 (SAS, Cary, NC).

## 3. Results

### 3.1. Demographic characteristics of HCPs (Table 1)

The mean age for participants was 34.7 ± 12.1 years, and 75 % were female. Approximately 49% of the respondents were doctors while nurses, dietitians/nutritionists, and pharmacists were 38 %, 4.2 %, and 9.4 %, respectively (Table 1). Majority of the HCPs were Christian (94.9 %) and received their professional training in Jamaica (80.6 %). Approximately 53 % of the HCPs had 5 years or less of work experience.

### 3.2. Knowledge, training, and personal use of CAM by HCPs (Table 2)

The overall prevalence of personal use of CAM was 43.4 %; this varied from approximately 39% among pharmacists and nurses to 45.6 % among doctors and 63.6% among dieticians (Table 2). Most of the HCPs (78.4%) reported little/some knowledge of CAM and less than half (47.7%) reported receiving training on CAM; 51.4 % of doctors, 44.2 % of nurses, 48.2% of pharmacists and 33.3% of dietitians/nutritionists reported receiving CAM training.

### 3.3. Recommendation of the use of CAM to patients and attitude towards use of CAM by HCPs (Table 3)

Sixty percent of HCPs reported that they were not likely to discuss CAM with patients and 66 % were not likely to recommend CAM to patients (Table 3). However, 41 % reported that they had recommended CAM use along with use of prescription medicine to their patients. Pharmacists and doctors (52.2 % and 49.2 %, respectively) were more likely to recommend CAM use along with prescription medicine to patients compared to nurses and nutritionists/dieticians (29.5 % and 16.7%, respectively; Table 3). Approximately 7% of HCPs believed that CAM is harmful and 7% believed that CAM does not work. Approximately 95% of HCPs said that they would not recommend their patients to an herbalist or traditional provider; 54.9% of HCPs were unlikely to recommend their patients to an alternative practitioner (Table 3).

### 3.4. Attitude of HCPs regarding the possible benefits of CAM use and the need for research and training of HCPs on CAM (Table 4)

A majority of HCPs (75.2 %) agreed that use of CAM and prescription medicine is better than use of prescription medicine alone, that HCPs need more education on CAM (86.6 %), and that CAM should be incorporated into health professions curricula (75.5 %; Table 4). Approximately 55 % of HCPs felt that the use of CAM and prescription medicine increases patient satisfaction and 61.9 % felt that CAM will enhance patient care. Over half of HCPs felt that CAM promotes health and wellness in patients with HTN (56.7 %) or T2DM (55.3 %) and can assist in fighting HTN (60.3 %) and T2DM (54.8 %). The overwhelming majority of HCPs (91.0 %) agreed that more research on the safety of CAM use for HTN and T2DM should be performed. A majority of the pharmacists (74–78 %) believed that CAM assists in fighting illness and promotes wellness and better health in patients with HTN and T2DM.

### 3.5. Odd Ratios and 95 % CIs for discussion, recommendation, and personal use of CAM by HCPs (Table 5)

We found positive significant associations between marital status (married vs single, OR = 2.42; 95 % CI = 1.24–4.71), type of profession (physicians vs nurses OR = 2.17; 95 % CI = 1.07–4.42; pharmacists vs nurses OR = 8.67; 95 % CI = 2.83–26.57), training on CAM (Yes vs No, OR = 2.36; 95 % CI = 1.26–4.42), personal use of CAM (Yes vs No, OR = 8.94; 95 % CI = 4.76–16.8) and discussion of CAM use with patients (Table 5). Compared to nurses, physicians and pharmacists had higher odds of discussing CAM with patients.

With regard to recommendation of CAM use, there were positive significant associations between marital status (married vs single OR = 2.30; 95 % CI = 1.16–4.54), training on CAM (Yes vs No, OR = 2.34; 95 % CI = 1.24–4.40), personal use of CAM (Yes vs No, OR = 6.74; 95 %CI = 3.54–12.83) and recommendation of CAM use (Table 5).

Type of profession (dietician vs nurses, OR = 4.56; 95 % CI = 1.16–17.86), training on CAM (Yes vs NO, OR = 2.90; 95 % CI = 1.69–4.97) and self-assessed knowledge of CAM (average vs little/some OR = 2.69; 95 % CI = 1.27–5.72, good vs little/some OR = 3.26; 95 % CI = 1.04–10.47) were significantly associated with personal use of CAM by HCPs (Table 5). Notably, receiving training on CAM is significantly associated with discussion, recommendation, and personal use of CAM by HCPs. Study country (Jamaica vs other, OR = 0.45; 95 %CI = 0.22–0.92) was associated with lower odds of personal use of CAM.

## 4. Discussion

The proportion of HCPs in the study who reported little or some knowledge of CAM (78.0%) was similar to the proportion who received low or average scores (81.0%) on the CAM knowledge test. Similarly, 50–75 % of HCPs in Trinidad and Tobago reported only fair knowledge of herbal, spiritual, alternative, and physical types of CAM.^21^ This indicates that a majority of HCPs in Jamaica and Trinidad and Tobago did not believe that they have good knowledge of CAM and that the Jamaican HCPs assessment of their knowledge of CAM was fairly accurate based on their scores on the CAM knowledge test.

Approximately 48% HCPs in this study reported receiving training on CAM indicating the need for HCPs to receive training on CAM during their professional programs. Although courses and/or modules in CAM are offered at institutions providing professional training to doctors and nurses in Jamaica, these courses/modules are designated for students in specialized aspects of particular programs and are not a part of the required curriculum for most students and could possibly be taken as electives by students. For example, students in the third year Bachelor of Medicine, Bachelor of Surgery (MBBS) program at the University of the West Indies (UWI) in Mona, Kingston are offered a module on CAM under the Community Health Clerkship Aspects of Family medicine. The UWI School of Nursing provides a few lectures on CAM to students. An Herbal Elective course and post-grad courses on CAM are offered to pharmacy students attending the University of Technology (UTECH) and courses in CAM are offered to students in nutrition and dietetics at Northern Caribbean University. No CAM course was explicitly listed in the 4-year B.Sc. in nutrition and dietetics curriculum at UTECH. A study on CAM in medical schools in the United States of America (USA) found that half of the schools (50.8%) offered at least one CAM course or clerkship.^31^ In these schools CAM is covered under a wide range of topics such as traditional medicine, acupuncture, spirituality, and herbs. A recent study that focused on CAM training among nurses, midwives and dieticians in Turkey found that only a few students (8.5 %) had received training on Complementary and Integrative Medicine (herbal remedies, acupuncture, aromatherapy, and massage).^32^ The majority of HCPs in the current study agreed that more education on CAM is necessary and that CAM should be incorporated into health professions training curricula. This could be accomplished through partnership of the Ministry of Health, the Western Regional Health Authority and health professions institutions in identifying, developing, and adding CAM to curricula provided during professional training.

Exercise and diet were the most common CAM methods used by the HCPs. This may be related to the fact that exercise and diet have long been shown to be the most effective methods for maintaining a healthy weight, thereby preventing the development of chronic diseases such as T2DM and cardiovascular diseases including HTN.^33,34^ HCPs do not have good evidence for the efficacy and safety of other commonly used CAMs such as herbal treatments, nutritional supplements, and manual techniques, and therefore recommend these methods less than exercise and diet.

The HCP group with the highest percentage of respondents that recommended CAM use along with prescription medicine to patients was pharmacists. Pharmacists also reported herbal medicine as the number one type of CAM that they used. This is in sharp contrast to doctors and nurses who reported exercise as their number one type of CAM and dieticians/nutritionists who reported nutritional supplement as their number one CAM. It may be that pharmacists feel more confident in their use of certain herbs and therefore recommended them to their patients. Garlic was the herb most commonly reported by all groups of HCPs; however, 70 % of pharmacists reported using garlic compared to 50–58 % of other groups of HCPs. A recent study conducted in the United States reported that pharmacists selectively used vitamins, minerals, herbals, and other dietary supplements and recommended some of the more commonly used products to patients.^35^

In the current study, physicians and pharmacists had two times and 8.7 times higher odds, respectively, of discussing CAM with patients than nurse but were not more likely to recommend CAM use to patients. These findings are consistent with the study conducted in Trinidad and Tobago where doctors, compared to other HCPs, were most likely to ask their patients about CAM use (67.5 %) but least likely to recommend CAM (26 %).^21^ The types of CAM investigated in Trinidad and Tobago (herbal, spiritual, physical, alternative, energy therapy and therapeutic methods) were similar to those investigated in our study. The findings from the two studies might reflect the varied and limited training and information on CAM that the HCPs received and their readiness or reluctance to discuss or recommend CAM based on their knowledge on the topic. Perhaps the dynamics between HCPs in different countries vary. Many of the nurses in our study stated that they deferred to the physicians’ suggestions. Therefore, a professional hierarchy may be followed. In addition, the standards of protocol expected by the governmental authority for which our HCPs worked may have influenced their responses.

Training on CAM was significantly associated with discussion and recommendation of CAM as well as personal use of CAM by HCPs. HCPs who reported receiving training on CAM were 2.36 times more likely to discuss CAM, 2.72 times more likely to recommend CAM and 2.90 times more likely to personally use CAM, compared to those who reported no training on CAM. These results may indicate that training on CAM provides HCPs with the knowledge and confidence to discuss and recommend CAM to their patients and also to use CAM themselves. Personal use of CAM by HCPs was also significantly associated with discussion and recommendation of CAM to patients. Thus, use of CAM by HCPs might provide them with familiarity and satisfying results that allow them to recommend CAM to their patients. Our findings on the personal use of CAM by 43.4 % of HCPs (45.6 % of doctors), and recommendation of CAM to patients by 34.1 % of HCPs (37.3 % of doctors) are not consistent with the study from Trinidad and Tobago where 65–92 % of HCPs (65 % of doctors) reported personal use of CAM, but only 26 % of doctors recommended CAM use to patients.^21^ This may reflect variation in the different cultural settings that dictate acceptability of recommending CAM.

Type of profession, training on CAM and self-assessed knowledge of CAM were significantly associated with personal use of CAM by HCPs. HCPs who self-assessed their knowledge of CAM as average or good had higher odds of personal use of CAM. Dieticians/nutritionists had higher odds for personal use of CAM compared to nurses. This may be due to the fact that unlike other professionals, some dieticians may be certified in CAM and that dieticians expect to receive training on CAM approaches to use in their practice.^36^ Dieticians/nutritionists may also continually seek out opportunities for evidence-based information, since their training is primarily acute care or disease prevention-focused.^36^ The type of CAM used by the overwhelming majority of dieticians/nutritionists was nutritional supplement (88.9 %); diet was their third most commonly reported CAM after exercise. Curricular training on CAM can be made applicable to all HCPs. Integrating CAM into the curricula of health professions schools can provide HCPs with knowledge of CAM allowing them to give appropriate counseling and care to their patients. Additionally, CAM training could be required as part of continuing education credits. As such, CAM training could be acquired and fostered on the job as well as by attending conferences or workshops where papers on CAM are presented or discussed.^14^

The Pan American Health Organization’s guidelines on Managing Hypertension in Primary Care in the Caribbean, indicate the need for greater public education, improved access to services, and greater cooperation between patient and HCPs to ensure adherence to treatment goals.^37^ Strategies for public education on CAM, for both patients and HCPs, should include information on the benefits and/or detriments of CAM. This can be achieved by documenting generational familiarities of herbal remedies and conducting research on their safety and appropriate doses. Several medicinal plants have been studied at the University of the West Indies in Jamaica over the years; natural products have been identified and some found to have medicinal properties.^38,39^ Some of these herbs could be incorporated into treatment plans with the support of strong scientific data.

Interestingly, a majority of HCPs (75.2 %) agreed that knowledge of CAM and prescription medicine is better than knowledge of prescription medicine alone. More than half of HCPs felt that the use of CAM and prescription medicine increases patient satisfaction, and that CAM will enhance patient care, promote health and wellness in patients with HTN or T2DM, and can assist in fighting these diseases. However, the majority of HCPs (87 %) felt that HCPs need more education on CAM and that more research on the safety of CAM use for HTN and T2DM should be performed. Therefore, it seems that until HCPs receive training on CAM and feel assured that recommendations regarding CAM use are safe and have scientific merit, they will be reluctant to discuss or recommend CAM use to patients and will continue to use conventional medications in treating HTN and T2DM.

### 4.1. Limitations

Several limitations should be considered in interpreting the results of this study. The first is that the small sample size for some categories of HCPs and incomplete responses to some questions could have prevented significant findings in the study. Second, the study sample represents only HCPs who treated patients with HTN and/or T2DM in chronic non-communicable disease clinics in the four parishes of Western Jamaica; therefore, the results may not be generalizable to HCPs in other clinics, private healthcare facilities, and other health regions of Jamaica. However, both public and private pharmacists in the Western Region were included. Third, the data were self-reported and might be subject to social desirability bias. Additionally, HCPs had different definitions for CAM which might have affected some of their responses, e.g., some did not believe that activities such as exercise and spirituality fell into the definition of CAM.

The limited number of types of CAM investigated could be considered a limitation of the study, however, the types of CAM that were studied were those that were more likely to be encountered in the public health sector in Jamaica where most patients with HTN and T2DM receive their medical care. Types of CAM provided by Chiropractic, Acupuncture, Homeopathic and Reiki practitioners were not included since these types of practitioners are not employed in the public health sector in Jamaica and most patients who receive care for HTN and T2DM in the public health system do not have the financial resources to access these types of practitioners. Training of 81.0 % of study participants in Jamaica may prevent generalization of our findings to other countries or regions of the world; however, since the institutions that train HCPs in Jamaica train students from 17 Caribbean countries, it is likely that our findings may be generalizable to these countries of the Caribbean.

## 5. Conclusions

Regardless of the limitations, this study identified several factors, such as type of profession and training in CAM, that are significantly associated with discussion, recommendation and use of CAM. These findings can be used to guide the development of CAM training programs for HCPs. The proportion of HCPs that assessed their knowledge of CAM as average or little/some is similar to the percentage that received poor or average scores on the CAM knowledge test. Thus, HCPs have a fairly accurate assessment of their knowledge on CAM and could benefit from training on CAM during and after their professional programs. The finding that a majority of HCPs believe that CAM is beneficial but were unlikely to discuss or recommend CAM to their patients identified a significant gap in open communication on CAM between HCPs and patients. CAM use by patients is extremely high and not addressing this issue could result in low adherence to prescribed treatment, less effective health outcomes and significantly poorer quality of life of patients living with these chronic diseases. However, the vast majority of HCPs feel that more education on CAM and more research on the safety of CAM use for HTN and T2DM are predominant needs. Interest in the integration of CAM into the Jamaican healthcare system is high and could be of great benefit to HCPs and their patients.

## Figures and Tables

**Table 1:** Demographic characteristics of healthcare professionals.

	Total N (%) 288 (100.0)	Doctors N (%) 141 (48.9)	Nurses N (%) 108 (37.5)	Dieticians/ Nutritionists N (%) 12 (4.2)	Pharmacists N (%) 27 (9.4)	P-value
Age (Mean ± SD)	34.7 (12.1)	33.1 (12.5)	34.9 (10.4)	43.1 (12.1)	39.4 (14.5)	0.0159
Gender						
Male	68 (24.6)	57 (40.7)	4 (4.0)	1 (10.0)	6 (23.0)	<.001
Female	208 (75.4)	83 (59.3)	96 (96.0)	9 (90.0)	20 (77.0)	
Marital Status						
Married	94 (33.6)	38 (27.1)	39 (38.2)	5 (45.5)	12 (44.5)	0.0026
Single	173 (61.8)	101 (72.2)	53 (52.0)	6 (54.5)	13 (48.1)	
Divorced/ Widowed	13 (4.6)	1 (0.7)	10 (9.8)	0 (0.0)	2 (7.4)	
Religion						
Christianity	244 (94.9)	111 (89.5)	96 (100.0)	11 (100.0)	26 (100.0)	0.0021
Others	13 (5.1)	13 (10.5)	0 (0.0)	0 (0.0)	0 (0.0)	
Parish						
St James	155 (56.0)	78 (55.7)	49 (49.0)	8 (66.7)	20 (80.0)	0.0222
Westmoreland	63 (22.7)	40 (28.6)	19 (19.0)	1 (8.3)	3 (12.0)	
Hanover	36 (13.0)	14 (10.0)	18 (18.0)	2 (16.7)	2 (8.0)	
Trelawny	23 (8.3)	8 (5.7)	14 (14.0)	1 (8.3)	0 (0.0)	
Years of experience						
<5years	152 (53.3)	91 (65.5)	49 (45.8)	3 (25.0)	9 (33.3)	0.0038
5–20	70 (24.6)	21 (15.1)	34 (31.8)	5 (41.7)	10 (37.1)	
21–30	45 (15.8)	18 (12.9)	18 (16.8)	4 (33.3)	5 (18.5)	
≥ 31	18 (6.3)	9 (6.5)	6 (5.6)	0 (0.0)	3 (11.1)	
Professional training						
Jamaica	232 (80.6)	96 (68.1)	97 (89.8)	12 (100.0)	27 (100.0)	<.0001
Outside Jamaica	56 (19.4)	45 (31.9)	11 (10.2)	0 (0.0)	0 (0.0)	

**Table 2:** Knowledge, training, and personal use of complementary and alternative medicine (CAM) by healthcare professionals (HCPs).

	Total N (%) 288 (100.0)	Doctors N (%) 141 (48.9)	Nurses N (%) 108 (37.5)	Dietician/ Nutritionist N (%) 12 (4.2)	Pharmacist N (%) 27 (9.4)	P-value
Training on CAM						
Yes	135 (47.7)	72 (51.4)	46 (44.2)	4 (33.3)	13 (48.1)	0.5170
No	148 (52.3)	68 (48.6)	58 (55.8)	8 (66.7)	14 (51.9)	
Self-reported knowledge of CAM						
Little/ some	221 (78.4)	116 (84.7)	81 (75.7)	11 (91.7)	13 (50.0)	0.0011
Average	42 (14.9)	15 (10.9)	20 (18.7)	0 (0.0)	7 (26.9)	
Good	19 (6.7)	6 (4.4)	6 (5.6)	1 (8.3)	6 (23.1)	
Knowledge score						
Poor (0–3)	89 (30.9)	53 (37.6)	30 (27.8)	1 (8.3)	5 (18.5)	0.0003
Average (4–6)	143 (49.7)	76 (53.9)	46 (42.6)	8 (66.7)	13 (48.2)	
Good (7–9)	56 (19.4)	12 (8.5)	32 (29.6)	3 (25)	9 (33.3)	
Source of CAM training						
School/college	110 (83.3)	63 (87.5)	39 (88.6)	2 (50.0)	6 (50.0)	0.0149
Work-related	13 (9.9)	5 (6.9)	3 (6.8)	1 (25.0)	4 (33.3)	
Literature/ patients	9 (6.8)	4 (5.6)	2 (4.6)	1 (25.0)	2 (16.7)	
Personal use of CAM						
Yes	121 (43.4)	62 (45.6)	42 (39.6)	7 (63.6)	10 (38.5)	0.3958
No	158 (56.6)	74 (54.4)	64 (60.4)	4 (36.4)	16 (61.5)	
Alternative treatment methods used						
Herbal medicine	60 (46.2)	23 (33.3)	27 (62.8)	2 (22.2)	8 (88.9)	0.0004
Nutritional supplement^a^	69 (53.1)	39 (56.5)	18 (41.9)	8 (88.9)	4 (44.4)	0.0601
Diet^a^	101 (77.7)	63 (91.3)	26 (60.5)	5 (55.6)	7 (77.8)	0.0006
Manual techniques^b^	12 (9.3)	7 (10.1)	2 (4.8)	2 (22.2)	1 (11.1)	0.4073
Exercise	105 (81.4)	64 (92.8)	28 (66.7)	6 (66.7)	7 (77.8)	0.0041
Spiritual healing	21 (16.3)	8 (11.6)	11 (26.2)	1 (11.1)	1 (11.1)	0.2131
Relaxing techniques^c^	38 (29.7)	13 (18.8)	17 (40.5)	3 (37.5)	5 (55.6)	0.0250

a Diet refers to the foods and beverages a person consumes. Nutritional supplements are any pill, capsule, tablet, liquid, or powder that contains vitamins, minerals, herbs, and/or amino acids intended to increase dietary intake of these substances.^40^

b Manual techniques refer to “therapy in which skilled hand movements are used to manipulate one or more parts of the patient’s body to treat pain, stress, anxiety, and depression, and for general well-being. Examples include chiropractic treatments, physical therapy, and massage therapy”.^41^.

c Relaxing techniques refer to various practices that can help to reduce stress, primarily meditation and biofeedback. Meditation is considered a type of mind-body complementary medicine that can produce a deep state of relaxation and a tranquil mind. Biofeedback is used to help manage many physical and mental health issues and can be used to learn how to control some of the body’s functions, such as heart rate and breathing.^42,43^.

**Table 3:** Discussion and recommendation of complementary and alternative medicine (CAM) by healthcare professionals (HCPs).

	Total N (%)	Doctors N (%)	Nurses N (%)	Dietician/ Nutritionist N (%)	Pharmacist N (%)	P-value
	288 (100.0)	141 (48.9)	108 (37.5)	12 (4.2)	27 (9.4)	
Discuss CAM use with patients						
Yes	113 (40.1)	58 (42.3)	32 (30.2)	5 (41.7)	18 (66.7)	0.0057
No	169 (59.9)	79 (57.7)	74 (69.8)	7 (58.3)	9 (33.3)	
Recommend CAM use to patients						
Yes	94 (34.1)	50 (37.3)	33 (31.7)	2 (16.7)	9 (34.6)	0.4748
No	182 (65.9)	84 (62.7)	71 (68.3)	10 (83.3)	17 (65.4)	
Recommend CAM use along with prescription medicine						
Yes	107 (40.8)	65 (49.2)	28 (29.5)	2 (16.7)	12 (52.2)	
No	155 (59.2)	67 (50.8)	67 (70.5)	10 (83.3)	11 (47.8)	0.0045
Most used herb for hypertension						
Garlic	160 (55.6)	71 (50.4)	63 (58.3)	7 (58.3)	19 (70.4)	0.1069
Ginger	4 (1.4)	1 (0.7)	1 (0.9)	0 (0.0)	2 (7.4)	
Lime	4 (1.4)	3 (2.1)	1 (0.9)	0 (0.0)	0 (0.0)	
Others	120 (41.6)	66 (46.8)	43 (39.9)	5 (41.7)	6 (22.2)	
HCPs report of the proportion of their patients who said they use CAM for hypertension						
0–25%	155 (60.1)	73 (57.5)	58 (61.7)	5 (41.7)	19 (76.0)	0.3651
26–50%	65 (25.2)	37 (29.1)	20 (21.3)	4 (33.3)	4 (16.0)	
51–100%	38 (14.7)	17 (13.4)	16 (17.0)	3 (25.0)	2 (8.0)	
HCPs report of the proportion of their patients who said they use CAM for type 2 diabetes						
0–25%	135 (64)	57 (57.6)	56 (69.1)	5 (50.0)	17 (81.0)	0.2201
26–50%	50 (23.7)	26 (26.3)	16 (19.8)	4 (40.0)	4 (19.0)	
51–100%	26 (12.3)	16 (16.1)	9 (11.1)	1 (10.0)	0 (0.0)	
HCP reaction to patient discussion of CAM use						
Use CAM instead of prescription medicine	5 (1.7)	3 (2.1)	2 (1.9)	0 (0.0)	0 (0.0)	
Use CAM along with prescription medicine	91 (31.6)	63 (44.7)	19 (17.6)	1 (8.3)	8 (29.6)	
Recommend they stop using CAM	76 (26.4)	34 (24.1)	36 (33.3)	3 (25.0)	3 (11.1)	
Ignore/not address the use of CAM	29 (10.1)	18 (12.8)	8 (7.4)	0 (0.0)	3 (11.1)	
Missing responses	87 (30.2)	23 (16.3)	43 (39.8)	8 (66.7)	13 (48.2)	
Recommendation of patients to herbalist/traditional practitioner by HCPs						
Yes	15 (5.4)	8 (5.8)	7 (6.7)	0 (0.0)	0 (0.0)	0.4630
No	265 (94.6)	129 (94.2)	98 (93.3)	12 (100.0)	26 (100.0)	
Reason for lack of recommendation						
Harmful	21 (7.4)	12 (8.5)	7 (6.5)	1 (8.3)	1 (3.7)	0.0275
Does not work	20 (6.9)	17 (12.1)	2 (1.9)	0 (0.0)	1 (3.7)	
Never considered	119 (41.3)	51 (36.1)	52 (48.1)	2 (16.7)	14 (51.9)	
No reason	128 (44.4)	61 (43.3)	47 (43.5)	9 (75.0)	11 (40.7)	
Likelihood of recommending to an alternative practitioner						
Likely	37 (13.3)	13 (9.7)	16 (15.4)	3 (25.0)	5 (18.5)	0.6031
Neutral	88 (31.8)	42 (31.3)	35 (33.7)	3 (25.0)	8 (29.6)	
Unlikely	152 (54.9)	79 (59.0)	53 (50.9)	6 (50.0)	14 (51.9)	

Traditional medicine refers to medical practices developed over generations within a society before the era of modern or conventional medicine. The World Health Organization (WHO) defines traditional medicine as “the sum total of the knowledge, skills, and practices based on the theories, beliefs, and experiences indigenous to different cultures, whether explicable or not, used in the maintenance of health as well as in the prevention, diagnosis, improvement or treatment of physical and mental illness”.^44^.

**Table 4:** The attitude of the healthcare professionals (HCPs) regarding the possible benefits of complementary and alternative medicine (CAM) use and the need for research and training of HCPs in CAM.

	Total N (%)	Doctors N (%)	Nurses N (%)	Dietician/ Nutritionist N (%)	Pharmacist N (%)	P-value
	288 (100.0)	141 (48.9)	108 (37.5)	12 (4.2)	27 (9.4)	
Use of CAM and prescription medicine is better than prescription medicine alone						
Agree	212 (75.2)	104 (75.9)	78 (73.6)	7 (58.3)	23 (85.2)	0.1229
Neutral	55 (19.5)	26 (19.0)	23 (21.7)	5 (41.7)	1 (3.7)	
Disagree	15 (5.3)	7 (5.1)	5 (4.7)	0 (0.0)	3 (11.1)	
Using CAM and prescription medicine increases patient satisfaction						
Agree	153 (54.5)	90 (65.7)	46 (43.4)	3 (27.2)	14 (51.9)	0.0017
Neutral	92 (32.7)	38 (27.7)	40 (37.7)	4 (36.4)	10 (37.0)	
Disagree	36 (12.8)	9 (6.6)	20 (18.9)	4 (36.4)	3 (11.1)	
HCPs need more education on CAM						
Agree	245 (86.6)	114 (83.2)	94 (87.9)	12 (100.0)	25 (92.6)	0.5426
Neutral	31 (11.0)	19 (13.9)	11 (10.2)	0 (0.0)	1 (3.7)	
Disagree	7 (2.4)	4 (2.9)	2 (1.9)	0 (0.0)	1 (3.7)	
CAM use should be incorporated into medical/nursing/nutrition/pharmacy curricula						
Agree	213 (75.5)	96 (70.6)	83 (77.6)	10 (83.3)	24 (88.9)	0.4161
Neutral	55 (19.5)	33 (24.3)	18 (16.8)	2 (16.7)	2 (7.4)	
Disagree	14 (5.0)	7 (5.1)	6 (5.6)	0 (0.0)	1 (3.7)	
CAM will enhance patient care						
Agree	174 (61.9)	84 (61.3)	62 (59.0)	6 (50.0)	22 (81.5)	0.2902
Neutral	93 (33.1)	47 (34.3)	36 (34.3)	6 (50.0)	4 (14.8)	
Disagree	14 (5.0)	6 (4.4)	7 (6.7)	0 (0.0)	1 (3.7)	
Research on CAM safety for hypertension and type 2 diabetes should be performed						
Agree	253 (91.0)	122 (90.4)	93 (89.4)	12 (100.0)	26 (96.3)	0.2469
Neutral	22 (7.9)	13 (9.6)	9 (8.7)	0 (0.0)	0 (0.0)	
Disagree	3 (1.1)	0 (0.0)	2 (1.9)	0 (0.0)	1 (3.7)	
CAM can assist in fighting hypertension in patients						
Agree	167 (60.3)	77 (57.0)	65 (62.5)	4 (36.4)	21 (77.8)	0.1339
Neutral	95 (34.3)	50 (37.0)	34 (32.7)	7 (63.6)	4 (14.8)	
Disagree	15 (5.4)	8 (6.0)	5 (4.8)	0 (0.0)	2 (7.4)	
CAM can assist in fighting type 2 diabetes in patients						
Agree	154 (54.8)	72 (52.9)	57 (53.8)	5 (41.7)	20 (74.1)	0.1038
Neutral	103 (36.7)	48 (35.3)	43 (40.6)	7 (58.3)	5 (18.5)	
Disagree	24 (8.5)	16 (11.8)	6 (5.6)	0 (0.0)	2 (7.4)	
CAM promotes health and wellness of patients with hypertension						
Agree	160 (56.7)	80 (58.4)	56 (52.8)	4 (33.3)	20 (74.1)	0.2287
Neutral	112 (39.7)	52 (38.0)	46 (43.4)	8 (66.7)	6 (22.2)	
Disagree	10 (3.6)	5 (3.6)	4 (3.8)	0 (0.0)	1 (3.7)	
CAM promotes better health and wellness in patients with type 2 diabetes						
Agree	156 (55.3)	78 (57.0)	54 (50.9)	4 (33.3)	20 (74.1)	0.1393
Neutral	113 (40.1)	51 (37.2)	48 (45.3)	8 (66.7)	6 (22.2)	
Disagree	13 (4.6)	8 (5.8)	4 (3.8)	0 (0.0)	1 (3.7)	
CAM use should be limited to patients with failed conventional therapy						
Agree	23 (8.1)	10 (7.2)	11 (10.4)	1 (8.3)	1 (3.7)	0.1202
Neutral	86 (30.3)	43 (30.9)	32 (30.2)	7 (58.4)	4 (14.8)	
Disagree	175 (61.6)	86 (61.9)	63 (59.4)	4 (33.3)	22 (81.5)	
Willing to allow patients to participate in CAM clinical trials						
Agree	161 (57.3)	84 (60.9)	55 (52.4)	9 (75.0)	13 (50.0)	0.0334
Neutral	99 (35.2)	47 (34.0)	42 (40.0)	3 (25.0)	7 (26.9)	
Disagree	21 (7.5)	7 (5.1)	8 (7.6)	0 (0.0)	6 (23.1)	

**Table 5:** Multivariate Logistic regression models of discussion, recommendation, and personal use of CAM by healthcare professionals.

	Discussion of CAM^a^		Recommendation of CAM^b^		Personal Use of CAM^c^	
	Odds Ratio	95 % CI	Odds Ratio	95 % CI	Odds Ratio	95 % CI
Marital status						
Married vs single	2.42	1.24–4.71	2.30	1.16–4.54		
Divorced/ widowed vs single	4.08	0.72–23.10	1.00	0.19–5.44		
Profession						
Physicians vs nurses	2.17	1.07–4.42	1.26	0.61–2.58	1.16	0.64–2.11
Dietician/nutritionist vs nurses	0.99	0.20–4.86	0.26	0.04–1.58	4.56	1.16–17.86
Pharmacist vs nurses	8.67	2.83–26.57	1.29	0.42–3.98	0.75	0.27–2.07
Training on CAM use vs no training	2.36	1.26–4.42	2.34	1.24–4.40	2.90	1.69–4.97
Self-assessed knowledge of CAM						
Average vs little/some	0.83	0.35–1.94	1.22	0.52–2.88	2.69	1.27–5.72
Good vs little/some	0.79	0.22–2.89	0.83	0.24–2.80	3.26	1.04–10.47
Personal use of CAM						
Yes vs no	8.94	4.76–16.8	6.74	3.54–12.83		
Study country						
Jamaica vs other			0.61	0.27–1.39	0.45	0.22–0.92

a Covariates include Marital status, Profession, Training on CAM use, Self-assessed knowledge and Personal use of CAM.

b Covariates include Marital status, Profession, Training on CAM use, Self-assessed knowledge and Personal use of CAM, study country.

c Covariates include Marital status, Profession, Training on CAM use, Self-assessed knowledge, study country.
